# *HyperKvasir*, a comprehensive multi-class image and video dataset for gastrointestinal endoscopy

**DOI:** 10.1038/s41597-020-00622-y

**Published:** 2020-08-28

**Authors:** Hanna Borgli, Vajira Thambawita, Pia H. Smedsrud, Steven Hicks, Debesh Jha, Sigrun L. Eskeland, Kristin Ranheim Randel, Konstantin Pogorelov, Mathias Lux, Duc Tien Dang Nguyen, Dag Johansen, Carsten Griwodz, Håkon K. Stensland, Enrique Garcia-Ceja, Peter T. Schmidt, Hugo L. Hammer, Michael A. Riegler, Pål Halvorsen, Thomas de Lange

**Affiliations:** 1 SimulaMet, Oslo, Norway; 2 Oslo Metropolitan University, Oslo, Norway; 3 University of Oslo, Oslo, Norway; 4 Department of Medical Research, Bærum Hospital, Bærum, Norway; 5 University of Bergen, Bergen, Norway; 6 Augere Medical AS, Oslo, Norway; 7 UIT The Arctic University of Norway, Tromsø, Norway; 8 Simula Research Laboratory, Oslo, Norway; 9 Department of Medicine (Solna), Karolinska Institutet, Stockholm, Sweden; 10 Cancer Registry of Norway, Oslo, Norway; 11 Klagenfurt University, Klagenfurt, Austria; 12 Medical Department, Sahlgrenska University Hospital-Mölndal, Mölndal, Sweden; 13 SINTEF Digital, Oslo, Norway; 14 Department of Medicine, Ersta hospital, Stockholm, Sweden

**Keywords:** Health care, Gastrointestinal diseases

## Abstract

Artificial intelligence is currently a hot topic in medicine. However, medical data is often sparse and hard to obtain due to legal restrictions and lack of medical personnel for the cumbersome and tedious process to manually label training data. These constraints make it difficult to develop systems for automatic analysis, like detecting disease or other lesions. In this respect, this article presents *HyperKvasir*, the largest image and video dataset of the gastrointestinal tract available today. The data is collected during real gastro- and colonoscopy examinations at Bærum Hospital in Norway and partly labeled by experienced gastrointestinal endoscopists. The dataset contains 110,079 images and 374 videos, and represents anatomical landmarks as well as pathological and normal findings. The total number of images and video frames together is around 1 million. Initial experiments demonstrate the potential benefits of artificial intelligence-based computer-assisted diagnosis systems. The *HyperKvasir* dataset can play a valuable role in developing better algorithms and computer-assisted examination systems not only for gastro- and colonoscopy, but also for other fields in medicine.

## Abstract (summary)

**Table:** 

Measurement(s)	lumen of digestive tract • lumen of colon
Technology Type(s)	Gastrointestinal Endoscopy • Colonoscopy
Sample Characteristic - Organism	Homo sapiens

Machine-accessible metadata file describing the reported data: 10.6084/m9.figshare.12759833

## Background & Summary

The human gastrointestinal (GI) tract is subject to numerous different abnormal mucosal findings ranging from minor annoyances to highly lethal diseases. For example, according to the International Agency for Research on Cancer (https://gco.iarc.fr/today/fact-sheets-cancers), the specialized cancer agency of the World Health Organization (WHO), GI cancer globally accounts for about 3.5 million new cases each year. These cancer types usually have combined mortality of about 63% and 2.2 million deaths per year^1–3^.

Endoscopy is currently the gold-standard procedure for examining the GI tract, but its effectiveness is considerably limited by the variation in operator performance^4–6^. This causes, for example, an average 20% polyp miss-rate in the colon^7^. Thus, improved endoscopic performances, high-quality clinical examinations, and systematic screening are significant factors to prevent GI disease-related morbidity and deaths. The recent rise of artificial intelligence (AI)-enabled support systems has shown promise in giving healthcare professionals the tools needed to provide quality care at a large scale^8,9^. The core of an efficient AI-based system is the combination of quality data and algorithms which teach a model to solve real-world problems like detecting pre-cancerous lesions or cancers in images. Today’s AI-based systems are predominantly using a subfield of AI called machine learning (ML), which usually requires training on thousands of data samples to perform well on any given task. However, health data is often sparse and hard to obtain due to legal constraints and structural problems in data collection. Nevertheless, an increasing number of promising AI solutions aimed for diagnostics in endoscopy^10–17^ are being developed. The datasets used for these systems, like CVC^18,19^ and the ASU-Mayo polyp database^20^, are rather small in the context of ML research. In other non-medical ML areas, datasets such as ImageNet^21^ consists of 14 million images. Table 1 gives an overview of all, to the best of our knowledge, existing datasets of images and videos from the human GI tract. As can be observed, they are rather small, and often limited to polyps. Several of these have also lately become unavailable.

**Table 1:** An overview of existing GI datasets.

Dataset	Findings	Size	Availability
CVC-356^18^	Polyps	356 images^†^	by request^●^
CVC-ClinicDB^19^ (also named CVC-612)	Polyps	612 images^†^	open academic
CVC-VideoClinicDB^18^ (also named CVC-12k)	Polyps	11954 images^†^	by request^●^
CVC-ColonDB^62^	Polyps	380 images^†*ψ*^	by request^●^
Endoscopy Artifact detection 2019^63^	Endoscopic Artifacts	5,138 images	open academic
ASU-Mayo polyp database^20^	Polyps	18,781 images^†^	by request^●^
ETIS-Larib Polyp DB^64^	Polyps	196 images^†^	open academic
KID^65^^◊^	Angiectasia, bleeding, inflammations, polyps	2371 images and 47 videos	open academic^●^
GIANA 2017^66^^◊^	Polyps & Angiodysplasia	3462 images and 38 videos	by request
GIANA 2018^67,68^^◊^	Polyps & Small bowel lesions	8262 images and 38 videos	by request
GASTROLAB^69^	GI lesions	Some 100s of images and few videos	open academic^♣^
WEO Clinical Endoscopy Atlas^70^	GI lesions	152 images	by request^♣^
GI Lesions in Regular Colonoscopy Data Set^71^	GI lesions	76 images^†^	by request
Atlas of Gastrointestinal Endoscope^72^	GI lesions	1295 images	unknown^●^
El salvador atlas of gastrointestinal video endoscopy^73^	GI lesions	5071 video clips	open academic^♣^
Kvasir^22^	Polyps, esophagitis, ulcerative colitis, Z-line, pylorus, cecum, dyed polyp, dyed resection margins, stool	8000 images	open academic
Kvasir-SEG^49^	Polyps	1000 images^†^	open academic
Nerthus^74^	Stool - categorization of bowel cleanliness	21 videos	open academic

^†^Including ground truth segmentation masks. ^◊^Video capsule endoscopy. ^●^Not available anymore. ^*ψ*^Contour.

^♣^Not really a dataset usable for machine learning. It is more a medical atlas or database for education with a several low-quality samples of various findings in the GI tract.

The images and videos in *HyperKvasir* were collected prospectively from routine clinical examinations performed at a Norwegian hospital from 2008 to 2016. We retrieved the images from the Picsara image documentation database (CSAM, Norway), a plug-in to the electronic medical record system, in 2016. As a first step, 4,000 of these images were labeled into eight different classes by medical experts and published as the Kvasir dataset^22^. The dataset was later extended to 8,000 images. Using Kvasir, researchers all over the world have started developing different ML models and AI systems for GI endoscopy^23–38^. Moreover, the Kvasir dataset has been used to organize international competitions, i.e., the Medico Task at MediaEval in 2017^39^ and 2018^40^ and the ACM Multimedia 2019 BioMedia Grand Challenge^41^.

Based on the lessons learned from publishing the Kvasir dataset and organizing competitions, it became clear that one of the biggest challenges in medical AI is still data availability. Data is hard to retrieve from the health care systems, approvals from medical committees are hard to get, medical experts have limited time, and there are no efficient tools to label such data. Therefore, with *HyperKvasir*, we significantly increase both the amount of labeled medical data for supervised learning and also release a large amount of unlabeled data. The new dataset contains 110,079 images and 374 videos from various GI examinations, resulting in 1 million images and frames in total. Regarding the value of unlabeled data, recent work in the ML community has shown remarkable improvements to tackle the challenge of lack of data. Instead of learning from a large set of annotated data, algorithms can now learn from sparsely labeled and unlabeled data. This technique is known as semi-supervised learning and has lately been successfully applied in different medical image analyses^42^. Examples of semi-supervised learning are self-learning^43,44^ and neural graph learning^45^, which both make use of unlabeled data in addition to a small number of labeled data to extract additional information^43,44,46^. We believe these new algorithms might be the development needed to make AI even more useful for medical applications. The unlabeled data of *HyperKvasir* is intended to be used in medical and technical communities to explore semi-supervised and unsupervised methods, and users of the data might even consider employing their own local experts to provide labels . Subsequently, in addition to the data description, we provide a baseline analysis using the labeled classes of the dataset and feasible future research directions for researchers interested in using the dataset.

## Methods

The image and video data were collected using standard endoscopy equipment from Olympus (Olympus Europe, Germany) and Pentax (Pentax Medical Europe, Germany) at the Department of Gastroenterology, Bærum Hospital, Vestre Viken Hospital Trust, Norway. Vestre Viken provides health care services to 490,000 people, of which 189,000 are covered by Bærum hospital. Parts of the collected data were annotated with class labels and segmentation masks. The annotations were done by at least one experienced gastroenterologist from Bærum hospital, the Cancer Registry of Norway or Karolinska University Hospital in Sweden, and one or more experienced persons working in the medical field such as a junior doctor or Ph.D. student. Though several physicians have assessed each labeled data record of the dataset, there is a chance that some of the assessments might be biased by the well-known observer variation, particularly regarding subtle changes like low-grade reflux esophagitis and ulcerative colitis. Such discrepancies have been demonstrated in previous studies^47,48^. To tackle this further, we decided to combine some of the findings that are prone to bias into one class (details about the classes and combinations can be found in the data records descriptions). Finally, a large number of unlabeled images are provided.

The study was approved by Norwegian Privacy Data Protection Authority and exempted from patient consent because the data were fully anonymous. All metadata was removed, and all files renamed to randomly generated file names before the internal IT department at Bærum hospital exported the files from a central server. The study was exempted from approval from the Regional Committee for Medical and Health Research Ethics - South East Norway since the collection of the data did not interfere with the care given to the patient. Since the data is anonymous, the dataset is publicly shareable based on Norwegian and General Data Protection Regulation (GDPR) laws. Apart from this, the data has not been pre-processed or augmented in any way.

### Class labels per image

The method for labeling images can be split into three distinct steps. First, experienced gastroenterologists involved in the project decided which classes should be included in the labeling process, based on medical relevance and the data collected. The selected classes were described in detail by medical experts. Second, two junior doctors or Ph.D. students working in the field annotated a subset of the images to the provided classes. Once this pre-labeling step was done, the medical experts checked the labels and adjusted when necessary. Cases where no consent could be found were discarded and replaced with new images from the dataset. The first dataset we created consisted of 4,000 images from eight classes^22^. This was later extended to 8,000 images for the same eight classes. For *HyperKvasir*, the dataset is further extended to 10,662 images and 23 classes. In total, *HyperKvasir* contains 110,079 images (10,662 labeled and 99,417 unlabeled images) from the GI tract.

### Segmentation masks per image

*HyperKvasir* includes images with corresponding segmentation masks and bounding boxes for 1,000 images from the polyp class. To create the segmentation masks, we uploaded 1,000 polyp images to the Labelbox platform (https://www.labelbox.com/). Labelbox is a tool that allows pixel-accurate labeling of image regions. First, a junior doctor and a Ph.D. student pre-segmented the 1,000 images. A gastroenterologist subsequently went trough all images verifying and correcting the pre-labeled segmentation masks. A detailed description of the annotation process and an example use-case of the dataset can be found in^49,50^.

### Descriptions per video

To get the labels per video, we uploaded the video data to a video annotation platform provided by Augere Medical AS (Oslo, Norway). Each video was analyzed and labeled by an experienced gastroenterologist. The class labels selected by the gastroenterologist were representing the main finding in the video as accurately as possible. For example, if the video contained footage of a polyp, the label for that video would be polyp. Additionally, there are examples of multiple findings in the same video. If so, these and more detailed descriptions are included in the *video-labeling.csv* file.

## Data Records

**Fig. 1:**
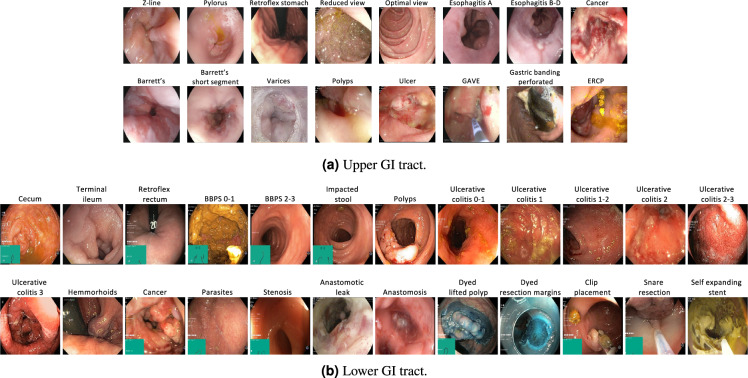
Image examples of the various labeled classes for images and/or videos.

**Fig. 2:**
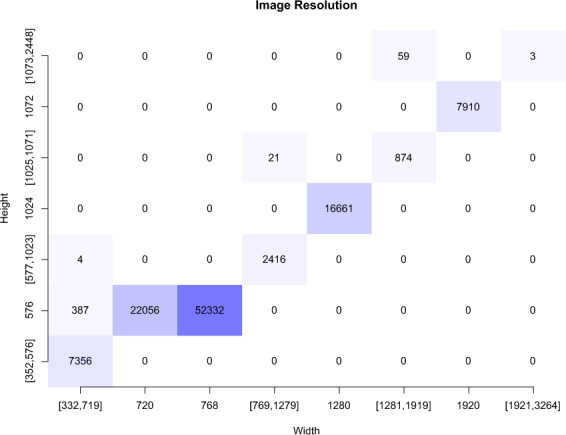
Resolution of the 110,079 images in *HyperKvasir*.

**Fig. 3:**
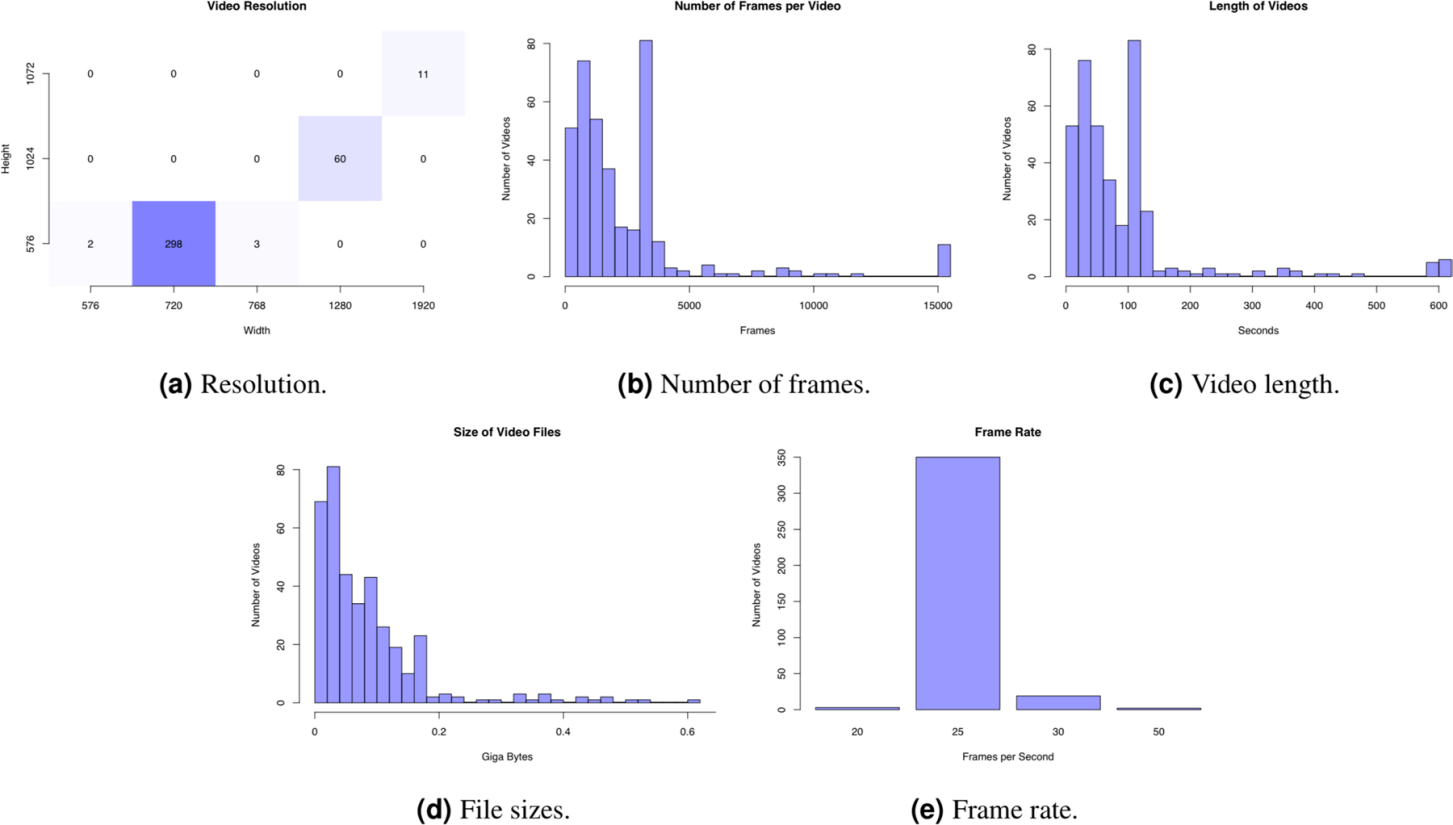
Statistics of the 374 videos in *HyperKvasir*.

The full *HyperKvasir*^51^ dataset, with all its images, videos and metadata, is available from the Open Science Framework (OSF) via the link 10.17605/OSF.IO/MH9SJ. The dataset is also available at https://datasets.simula.no/hyper-kvasir. *HyperKvasir* is open access and licensed under a Creative Commons Attribution 4.0 International (CC BY 4.0). In total, the dataset consists of four main data records. The records are labeled images, segmented images, unlabeled images, and labeled videos. All the various labeled classes are shown in Fig. 1, i.e., 16 classes from the upper GI tract (Fig. 1a) and 24 classes from the lower GI tract (Fig. 1b). The dataset has a size of circa 66.4GB (not including metadata files and segmentation masks), 32.5GB for videos and 33.9GB for images. An overview of all data records in the dataset is given in Table 2. Some of the images and videos contain a picture in picture (green thumbnail in the lower left corner) which represents the Olympus ScopeGuide^TM^ (Olympus Europe, Germany), used by the endoscopist to get a topographic view of the colon. Details about image and video resolutions and video frame rates can be found in the Figs. 2 and 3. The following subsections provide additional details for each data record.

**Table 2:** Overview of the data records in the *HyperKvasir* dataset.

Data Record	# Files	Description	Size (MB)
Labeled images	10,662 images	23 classes of findings	3,960
Segmented Images	1,000 images	Segmentation masks for polyp findings	57
Unlabeled Images	99,417 images	Unlabeled	29,940
Videos	374 videos	30 different classes	32,539

### Labeled images

**Fig. 4:**
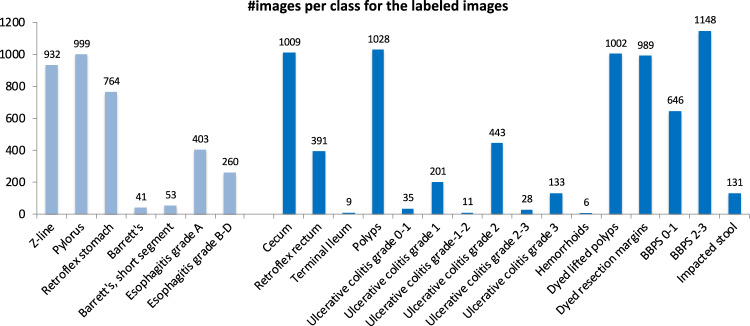
The number of images in the various *HyperKvasir* labeled image classes according to the file folders.

**Fig. 5:**
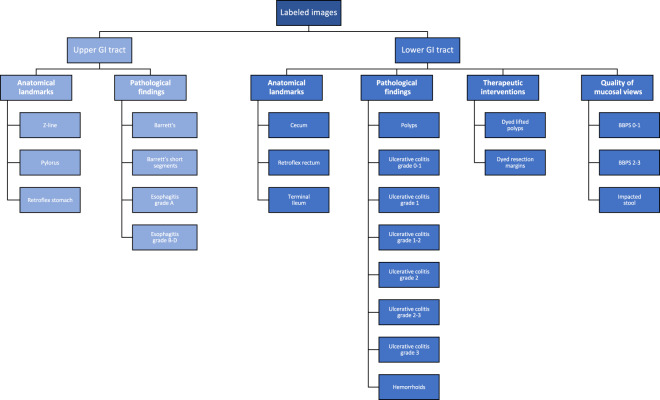
The various image classes structured under position and type, also the structure of the stored images.

In total, the dataset contains 10,662 labeled images stored using the JPEG format, where Fig. 4 shows the 23 different classes representing the labeled images and the number of images in each class. A CSV file is provided (image-labels.csv) giving the mapping between the image (file name) and the labeling for each image. These classes are structured according to location in the GI tract and the type of finding as shown in Fig. 5. We defined four main categories of findings where the first and the third are found both in the upper an lower GI tract:

- **Anatomical landmarks:** Anatomical landmarks are characteristics of the GI tract used for orientation during endoscopic procedures. Furthermore, they are used to confirm a complete extent of the examination. Landmarks exist both in the upper GI tract (esophagus, stomach and duodenum) and in the lower GI tract (terminal ileum, colon and rectum). However, in the small bowel, there are no specific landmarks to be used for topographical localization of a lesion.
- **Quality of mucosal views:** Complete visualization of all the mucosa is crucial not to overlook pathological findings. In the colon, there exist a classification for the quality of the mucosal vizualisation, the Boston Bowel Preparation Scale (BBPS)^52^.
- **Pathological findings:** All parts of the gastrointestinal tract can be affected by abnormalities or findings due to disease. Most pathological findings can be seen as more or less obvious changes in the intestinal wall mucosa. These findings are classified according to the Minimal Standard Terminology, defined by the World Endoscopy Organization^53^.
- **Therapeutic interventions:** When a lesion or pathological finding is detected, a therapeutic intervention is frequently required to treat the condition, e.g., lifting and resecting a polyp, dilation of a stenosis or injection of a bleeding ulcer.

Each class and the images belonging to it is stored in the corresponding folder of the category it belongs to. For example, the ’polyp’ folder in the category pathological findings in the lower GI tract contains all polyp images, the ’barrett’s’ folder in the category pathological findings in the upper GI tract contains all images of Barrett’s esophagus, etc. As observed in Fig. 2, the number of images per class are not balanced, which is a general challenge in the medical field due to the fact that some findings occur more often than others. This adds an additional challenge for researchers, since methods applied to the data should also be able to learn from a small amount of training data. Below, we detail each class further.

#### Upper Gastrointestinal tract

The upper GI tract examined by endoscopy includes the esophagus, stomach, and duodenum. Below, we give a description of the various classes of findings found here.

As seen in Fig. 5, we have labeled three classes of *anatomical landmarks* in the upper GI tract. The normal **Z-line** is the anatomical junction between the squamous epithelium of the esophagus and columnar epithelium of the stomach. A normal Z-line is located at the same level as the gastroesophageal junction. **Retroflex stomach** means that the endoscope is retroflexed, looking back to visualize the cardia and fundus in the upper parts of the stomach. The **pylorus** is the anatomical junction between the stomach and duodenal bulb, and a sphincter regulating the emptying process of the stomach into the duodenum.

All the following classes are defined as *pathological findings* in the upper GI tract. Reflux esophagitis is an inflammation mostly affecting the lower third of the esophagus, near the Z-line. Reflux esophagitis can be graded according to the Los Angeles (LA) classification^54^. The esophagitis LA classification is defined into four classes as (A) mucosal breaks shorter than 5mm, without continuity across mucosal folds where subtle changes can be difficult to differentiate from a normal Z-line; (B) mucosal breaks longer than 5mm that does not extend between the tops of two mucosal folds; (C) one (or more) mucosal break that is continuous between the tops of two or more mucosal folds, but which involves less than 75% of the circumference; and (D) one (or more) mucosal break that is continuous between the tops of two or more mucosal folds and involves more than 75% of the circumference. We have split esophagitis into two classes because there exists an important observer variation in the assessment of low grade esophagitis^47^. The two classes are **esophagitis A** and **esophagitis B-D**. This binary classification of the images makes it possible to assess whether mis-classification between normality and esophagitis only concern grade A. Barrett’s esophagus represents a metaplastic transformation of the squamous epithelium of the esophagus into a gastric like columnar epithelium. Barrett’s esophagus is considered a premalignant condition, meaning it might develop into cancer. Biopsies showing the presence of specialized intestinal metaplasia confirms the diagnosis. Barrett’s esophagus can be graded according to the Prague classification, describing the circumferential and longitudinal extension of the disease^55^. We have split the images of Barrett’s esophagus into two classes. **Barrett’s** long-segment and **Barrett’s, short-segment** esophagus where a short segment is characterized by a longitudinal extension of less than 3 cm^55^.

#### Lower gastrointestinal tract

The lower GI tract examined by colonoscopy includes the terminal ileum (last part of the small bowel), the colon and the rectum (the large bowel). Below, we describe the classes of the lower GI tract in the dataset.

We have labeled three classes of *anatomical landmarks* in the lower GI tract. The ileum is the distal 2/3 of the small bowel, recognized by visible intestinal villi. Endoscopically, the ileum cannot be distinguished from other parts of the small bowel. During colonoscopy, the distal 5–20 cm of the ileum, named **terminal ileum**, can be reached and examined. The visualization of the terminal ileum confirms complete colonoscopy. **Cecum** is the proximal end of the large bowel and is characterized by the visualization of the appendiceal orifice and the ileo-cecal valve. Complete examination of the whole colon can only be confirmed if the medial wall of the cecum has been visualized, that is the area between the appendiceal orifice and the ileo-cecal valve. The most distal part of the rectum is one of the blind zones of the colon. Therefore, the endoscope is retroflexed in the rectum to visualize the dentate line and the circumference of the proximal orifice of the anal canal, which is called **retroflex rectum**.

The *quality of the mucosal views* is a key quality indicator and should always be evaluated because a clean bowel is essential to detect pathological findings. In this respect, the degree of bowel cleansing during a colonoscopy is described by the Boston Bowel Preparation Scale (BBPS)^56^. BBPS consists of four different degrees which are: (BBPS 0) unprepared colon segment with no mucosa seen due to solid stool that cannot be cleared; (BBPS 1) portions of the mucosa of the colon segment seen, but other areas of the colon segment not well seen due to staining, residual stool and/or opaque liquid; (BBPS 2) minor amount of small fragments of stool and/or opaque liquid, but mucosa of colon segment seen well; and (BBPS 3) entire mucosa of colon segment seen well with no residual fragments of stool or opaque liquid. The bowel cleansing is deemed adequate if the BBPS score is 2 or 3 in all three segments of the colon after flushing. Therefore, we have labeled our images into the two **BBPS 0-1** and **BBPS 2-3** classes where class 0–1 represents inadequate bowel preparations, and the class 2–3 represents adequate bowel preparation. Moreover, a frequent finding in persons above the age of 50 years are pockets in the colon wall called diverticula and if numerous called diverticulosis. Sometimes stool is impacted in these diverticula and may increase the risk of diverticulitis. In the dataset, this is presented in the **impacted stool** class.

The following classes are defined as *pathological findings* in the lower GI tract. Ulcerative colitis is a chronic inflammatory bowel disease often debuting in the twenties. The degree and extent of the disease is determined by colonoscopy and can be classified according to the Mayo Score^57^. The Mayo Score for ulcerative colitis is defined: (Score 0) inactive, where the mucosa only has normal vascular patterns; (Score 1) mild with erythema, decreased vascular pattern, mild friability; (Score 2) moderate with erythema, absent vascular pattern, mild friability, erosions; and (Score 3) severe with spontaneous bleeding and ulcerations. For ulcerative colitis, we provide six different labeled classes, both the Mayo Score classes (**Ulcerative colitis 1/2/3**) and some classes in-between where it is difficult to determine the exact class and because previous studies have shown important observer variation in the assessment of the degree of inflammation (**Ulcerative colitis 0-1/1-2/2-3**)^48^. **Polyps** are most frequently neoplastic lesions of the large bowel. They have mainly three different shapes; protruding in the lumen, flat or excavated according to the Paris Classification^58^. Their size vary from 1 mm to several cm. The prevalence increases with age. The most common types of polyps are premalignant and can transform into cancer. Thus, it is important to discover polyps and remove the suspicious polyps during endoscopy. **Hemorrhoids** are pathologically swollen veins in the anus or lower rectum. When present in the rectum, they are called internal hemorrhoids, and when found in the anus, they are called external hemorrhoids.

Finally, *therapeutic interventions* show treatments of detected pathological findings. It includes for example lifting and removal of neoplastic tissue (polyps) and injection therapy of bleeding ulcer. The **dyed lifted polyps** class contains images of polyps lifted with submucosal injection using a solution containing indigo carmine. This is done prior to polyp resection for better diagnosis and easier resection. The dye is recognized by the blue color underneath the polyp. After resection of dyed polyps with a snare, the resection margins and site appears blue due to the indigo carmine solution. Images of these type of resection margin are presented in the **dyed resection margins** class.

### Segmented images

For the set of segmented images, we provide the original image, a segmentation mask and a bounding box for 1,000 images from the polyp class. In the mask, the pixels depicting polyp tissue, the region of interest, are represented by the foreground (white mask), while the background (in black) does not contain polyp pixels. The bounding box is defined as the outermost pixels of the found polyp. For this segmentation set, we have two folders, one for images and one for masks, each containing 1,000 JPEG-compressed images. The bounding boxes for the corresponding images are stored in a JavaScript Object Notation (JSON) file. The image and its corresponding mask have the same filename. The images and files are stored in the segmented images folder. It is important to point out that the images of polyp class from the Kvasir dataset had duplicates in the images folder. These duplicates were replaced by high-quality polyp images from the colon and segmented.

### Unlabeled images

In total, the dataset contains 99,417 unlabeled images. The unlabeled images can be found in the unlabeled folder which is a subfolder in the image folder, together with the other labeled image folders. In addition to the unlabeled image files, we also provide the extracted global features and possible unsupervised clustering assignments in the *HyperKvasir* Github repository as Attribute-Relation File Format (ARFF) files. ARFF files can be opened and processed using, for example, the WEKA machine learning library, or they can easily be converted into Comma-Separated Values (CSV) files.

### Labeled videos

**Fig. 6:**
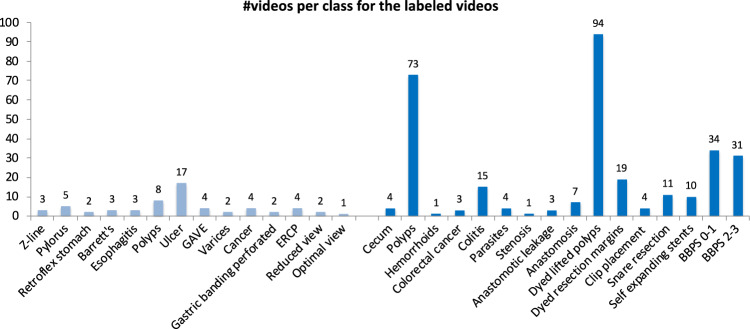
The number of videos in the various *HyperKvasir* labeled video classes according to the file folders.

**Fig. 7:**
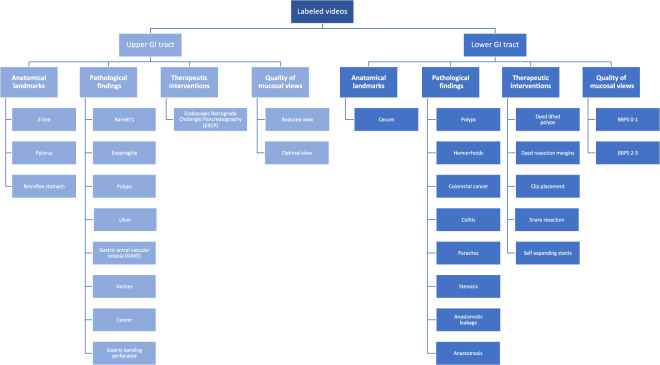
The various video classes structured under position and type, which is also the structure of the video folders.

The labeled videos are recorded for clinical purposes and thus represent daily practice. In total, 374 videos are provided in the dataset, which correspond to 9.78 hours of videos and 889,372 video frames that can be converted to images if needed. In total, an experienced gastroenterologist have identified 30 classes of findings, and Fig. 6 shows how many videos we have identified for each class. The class describes the video as a whole using the main finding, but additionally, many videos include more than one category and several classes where, for example, a single video can contain polyps, dyed lifted polyps and dyed resection margins. The video file format is Audio Video Interleave (AVI), and they are stored in the folder called labeled videos. As seen in Fig. 7, the videos are further organized and stored according to either upper or lower GI tract and then the four main categories as for the labeled images described above. In addition to the video files, a CSV file is provided (video-labels.csv) containing the videos’ *videoID* and *labeling*. Here, the VideoID contains the corresponding video file name, and the labeling includes the upper or lower location, the category and the class with some detailed descriptions of the video. Below, we describe the new classes per category for the in total 60 videos from the upper GI tract and the 60 videos from the lower GI tract.

#### Upper Gastrointestinal tract

As seen in Fig. 7, we have many of the same classes for videos and for images, but since we have labeled all our videos, more classes are added for both the upper and lower GI-tract. In the upper GI tract, the three classes of *anatomical landmarks* (**Z-line**, **Pylorus** and **Retroflex stomach**) are described in the section for labeled images above. In the category of *pathological findings*, both **Barrett’s** esophagus and **esophagitis** are also described above, but here we also added some new classes. The first is **polyps** where the description above of polyps in the colon is also valid for the upper GI-tract. In addition, five new classes not previously described are included. Mucosal **ulcers** are quite common in the upper GI tract. Ulcers are nearly always caused by Helicobacter pylori infection, ulcerogenic medication, or cancer. Ulcers are characterized according to the Forrest classification to predict the risk of bleeding^59^. Forrest I represents ongoing bleeding, Forrest II presents some signs of previous bleeding; and Forrest III does not show any sign of bleeding. The second class **Gastric antral vascular ectasia (GAVE)** represents dilated small superficial vessels in the mucosa of the gastric antrum. These lesions may cause chronic bleeding and subsequent anemia and are frequently treated by argon plasma coagulation (APC) to prevent further bleeding. **Varices** (dilated veins) in both the esofagus and the fundus of the stomach are most frequently caused by chronic liver diseases complicated with liver cirrhosis. The varices represent a major risk for severe bleeding. **Cancer** of the esophagus and the stomach are common findings in the upper GI-tract. The last class **gastric banding perforated** shows a rare finding, which is the complication of previous gastric banding operation where the band perforates the wall of the stomach. The category of *therapeutic interventions* are introduced for the Upper GI-tract especially because they are nearly always best illustrated by videos and can also serve important educational purposes. Since most of the *therapeutic interventions* are presented as secondary to a pathological finding we only include **Endoscopic Retrograde Choleangio-Pancreatografi (ERCP)** a procedure to treat gall-duct abnormalities as an independent class. However, other common therapeutic interventions such as the two thermal methods; APC and heatherprobe as well as injection therapy with adrenaline and application of hemospray to stop bleeding can be found under second findings in the csv file. In the category *quality of mucosal view*, we also added a footage showing **reduced view** due to opaque liquid in the stomach or air bubbles in the duodenum. Reduced view increases the risk of missing lesions. In opposite, **optimal view** demonstrates excellent visualization of the duodenum.

#### Lower Gastrointestinal tract

The videos from the lower GI tract illustrate mainly the same categories and classes as the labeled images. Nevertheless, they increase the diversity of the dataset. The category *anatomical landmarks* differs from the labeled images as it only contains the **cecum** class and does not include the classes of terminal ileum and retroflex rectum, only defined as second findings. The two categories pathological findings and therapeutic intervention also are a bit different compared to the labeled images. In the category *pathological findings*, we still have the above described **polyps** and **hemorrhoids** classes. However, all classes of ulcerative colitis are merged to **colitis** and also includes ischemic colitis and infectious colitis. The new class **colorectal cancer**, the second most deadly cancer worldwide^60^, was added. Colorectal cancer may present itself in different ways in the colon, from tiny lesions with a diameter of 1 cm to larger tumors obstructing the entire lumen of the bowel and cover bowel segments of several centimeters. Moreover, **parasites**, a common finding of small worms moving around in the colon, are more often encountered in tropical areas. **Stenosis** is characterized by a narrow obstruction of the bowel caused either by inflammation or malignant diseases. Large neoplastic lesions like cancers are surgically resected and subsequently an **anastomosis** is made to restore normal bowel function. The anastomosis can be visualized during follow-up colonoscopies. A feared complication after large bowel surgery is **anastomotic leakage**, potentially causing smaller or larger cavities of anastomotic leak especially in the rectum. The last decade mini-invasive endoscopic *therapeutic interventions* has to some extent replaced traditional and laparoscopic surgery regarding the treatment of large polyps and stenosis of the colon. The classes **dyed lifted polyp** and **dyed resection margin** are described under labeled images but videos improve the illustration of the technique. Three new classes are presented showing removal of polyps by simple **snare resection** or endoscopic mucosal resection (EMR). To prevent or stop bleeding after these resections, **clip placement** of metallic clips are illustrated. **Self expanding stents** are used to open and dilate either benign or malignant stenosis. Finally, in the *quality of mucosal views* category, we have removed the impacted stool class we have for images, and include only the above described **BBPS 0-1** and **BBPS 2-3** classes. Here, it is also worth noting that many of the videos in BBPS 2-3 are perfectly clean (BBPS 3), i.e., as then described in the csv-file, these contain videos of normal mucosa (also marked as finding 2) which can be extracted in normal images or videos when needed.

## Technical Validation

To demonstrate the technical quality of the dataset, we performed multiple experiments to provide some baseline metrics and to give some insight into the dataset’s statistical qualities. If the reader wants information about classification and segmentation approaches and experiments comparing state of the art methods using parts of this dataset, the reader is referred to other studies^49^.

### Baseline for supervised image classification

The presented dataset is suited for a variety of different tasks, one of which is image classification. As a preliminary step to evaluate how state-of-the-art methods perform on the labeled part of *HyperKvasir*, we performed a series of experiments based on methods that have previously achieved good results on GI tract image classification. The purpose of these experiments is merely to give example baseline results to be used by future researches to compare and measure their results. In total, we ran five experiments using different methods. The methods were primarily selected from the best performing methods presented at the MediaEval Medico Task^39,40^. Each method is based on deep convolutional neural networks, which is currently state-of-the-art within image classification. Common for all experiments is that the images were resized to 224 × 224 before being fed into the networks. All networks are based on common architectures, slightly modified to accommodate our task of classifying 23 different classes of images. The specifics of each method is further explained below:

- *Pre-Trained ResNet-50* is a TensorFlow implementation of the ResNet-50 architecture using ImageNet initialized weights. The network was trained in two steps. First, an initial training over 7 epochs, and then a fine-tuning step over 3 epochs which only trained the layers after the 100th index. Images were loaded using a batch size of 32, and the weights were optimized using Adam with a learning rate of 0.001.
- *Pre-Trained ResNet-152* is a PyTorch implementation of the ResNet-152 architecture using ImageNet initialized weights. The network was trained over 50 epochs using a batch size of 32, and optimized using Stochastic gradient descent (SGD) with a learning rate of 0.001. No fine-tuning was used for this method.
- *Pre-Trained DenseNet-161* is a PyTorch implementation of the standard DenseNet-161 architecture using ImageNet initialized weights. The network was trained over 50 epochs using a batch size of 32, and optimized using SGD with a learning rate of 0.001. No fine-tuning was used for this method.
- *Averaged ResNet-152* + *DenseNet-161*^38,61^ is an approach that combines the ResNet-152 and DenseNet-161 approach by averaging the output of both models as the final prediction. Both models were trained simultaneously by backpropagating the averaged loss through both models. Overall, the networks were trained for 50 epochs using a batch size of 32. SGD was used to optimize the weights with a learning rate of 0.001. Both the ResNet-152 and DenseNet-161 models were initialized using the best weights of the above Pre-Trained ResNet-152 and Pre-Trained DenseNet-161 implementations.
- *ResNet-152* + *DenseNet-161* + *MLP*^38,61^ is similar to the previous method using both ResNet-152 and DenseNet-161 to generate a prediction. However, instead of averaging the output of each model, this method uses a simple multilayer perceptron (MLP) to estimate the best way to average the output of each model. All networks were trained simultaneously over 50 epochs using a batch size of 32. The weights were optimized using SGD with a learning rate of 0.001. Both the ResNet-152 and DenseNet-161 models were initialized using the best weights of the above two implementations of Pre-Trained ResNet-152 and Pre-Trained DenseNet-161.

**Fig. 8:**
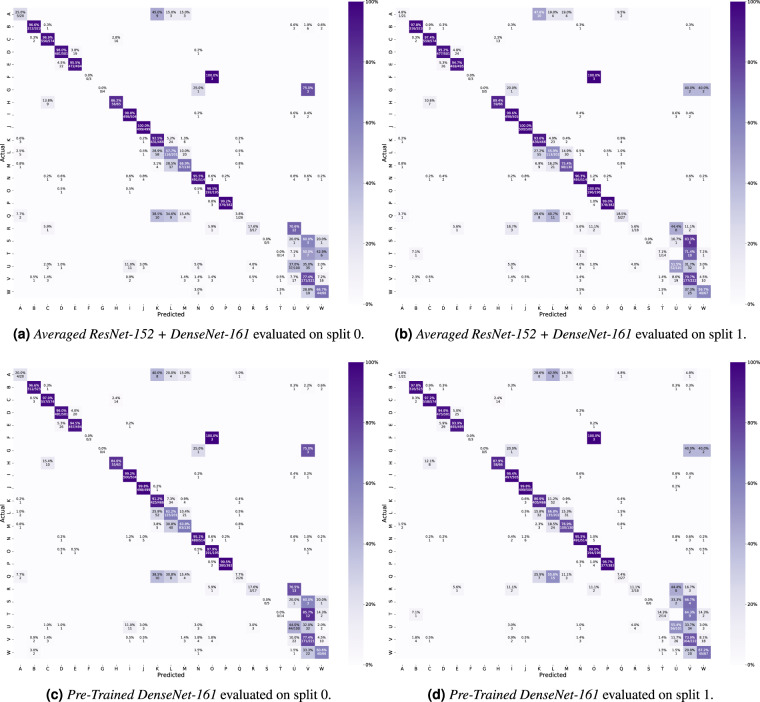
Confusion matrices for Averaged ResNet-152 + DenseNet-161 and Pre-Trained DenseNet-161 including both splits. These confusion matrices were selected based on their performance. Averaged ResNet-152 + DenseNet-161 achieved the best micro-averaged results while the Pre-Trained DenseNet-161 achieved the best macro-averaged result. The color codes represent the percentages of the total number of images within each class. The labeling of the classes is as follows: (A) Barrett’s; (B) bbps-0-1; (C) bbps-2-3; (D) dyed lifted polyps; (E) dyed resection margins; (F) hemorrhoids; (G) ileum; (H) impacted stool; (I) normal cecum; (J) normal pylorus; (K) normal Z-line; (L) oesophagitis-a; (M) oesophagitis-b-d; (N) polyp; (O) retroflex rectum; (P) retroflex stomach; (Q) short segment Barrett’s; (R) ulcerative colitis grade 0-1; (S) ulcerative colitis grade 1-2; (T) ulcerative colitis grade 2-3; (U) ulcerative colitis grade 1; (V) ulcerative colitis grade 2; (W) ulcerative colitis grade 3.

Each method was evaluated using standard classification metrics including the macro-averaged and micro-averaged F1-score, precision, and recall. Additionally, we calculated the Matthews correlation coefficient (MCC) for each experiment using the multi-class generalization which is also known as the *R*_*K*_. The results in Table 3 show that each method beats the random and majority class baseline by a large margin. However, the presented numbers also indicate that there is room for improvement. Looking at the confusion matrices in Fig. 8, we see that some classes are harder to identify than others. For example, there is a lot of confusion surrounding the difference between the grades of ulcerative colitis and esophagitis. Furthermore, there is also some confusion between specific classes such as dyed lifted polyps and dyed resection margins, and distinguishing Barrett’s from esophagitis or a normal Z-line. At least the confusion between classes of Z-line, esophagitis and Barrett’s esophagus is similar to the human variation in the assessment of these lesions. Thus, it is challenging to create a ground truth.

**Table 3:** Average results for the five tested classification approaches, i.e., average of the results for the two splits.

Method	Macro Average			Micro Average			
	Precision	Recall	F1-score	Precision	Recall	F1-score	MCC (*R*_*K*_)
Pre-Trained ResNet-50	0.589	0.536	0.530	0.839	0.839	0.839	0.826
Pre-Trained ResNet-152	0.639	0.605	0.606	0.906	0.906	0.906	0.898
**Pre-Trained DensNet-161**	0.640	0.616	0.619	0.907	0.907	0.907	0.899
**Averaged ResNet-152 + DenseNet-161**	**0.633**	**0.615**	**0.617**	**0.910**	**0.910**	**0.910**	**0.902**
ResNet-152 + DenseNet-161 + MLP	0.612	0.606	0.605	0.909	0.909	0.909	0.902
Random Guessing	0.044	0.038	0.034	0.044	0.044	0.044	0.000
Majority Class	0.004	0.043	0.008	0.108	0.108	0.108	N/A

### Composition of unlabeled data

**Fig. 9:**
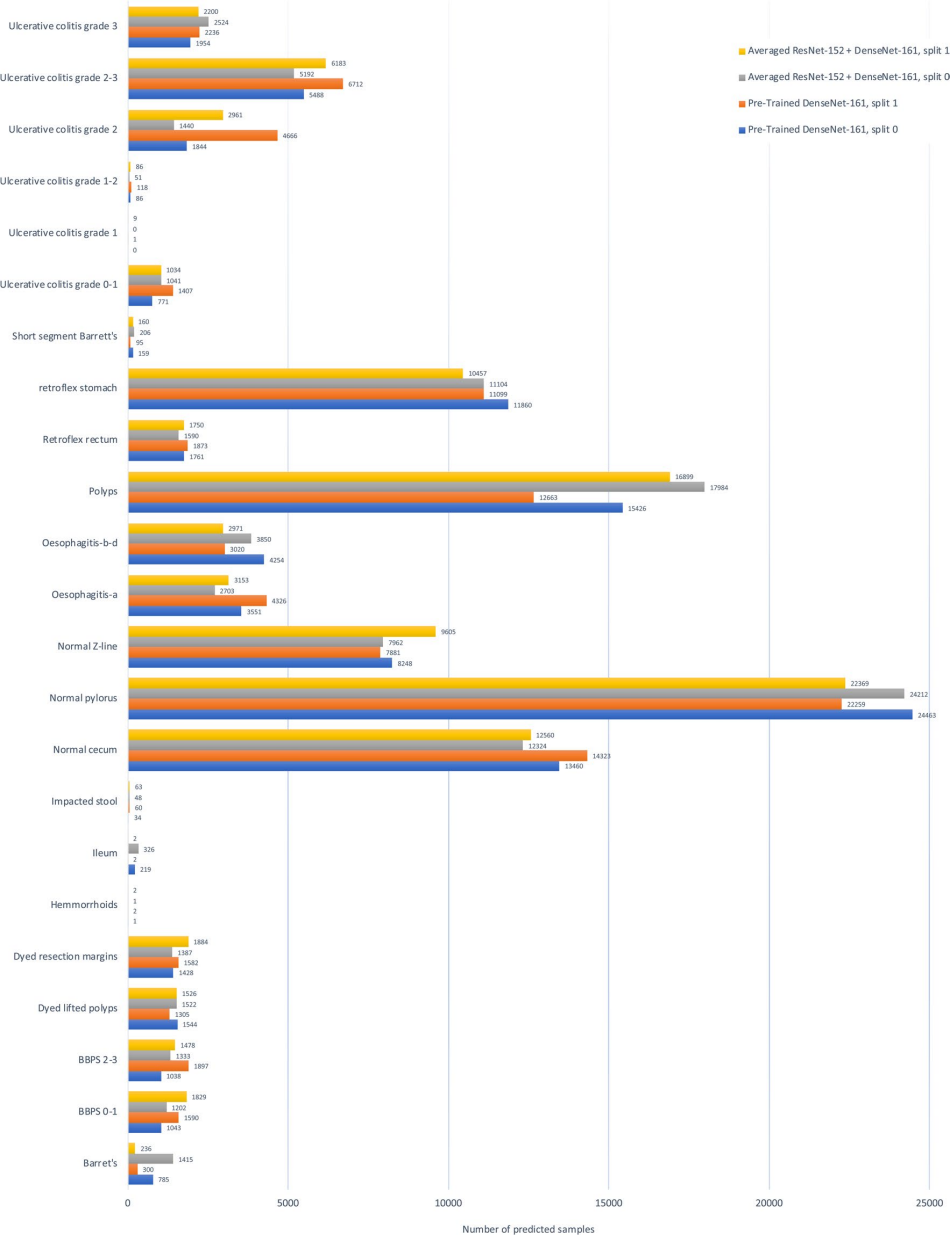
Unlabeled image data predictions for *Averaged ResNet-152* + *DenseNet-161* and *Pre-Trained DenseNet-161*.

In order to show the approximate composition of the unlabelled data, we present some initial experiments to analyze the provided data which do not have annotated labels from medical experts We used our pre-trained classification model to simply classify the unlabeled data to indicate how many of the labeled classes are in the unlabeled data and to get an overall idea about data distribution of the 99,417 images. In particular, we used the best two classification models from the previous experiments, i.e., Pre-Trained DenseNet-161 and Averaged ResNet-152 + DenseNet-161 using split_0 and split_1 from the previous experiment. The result are shown in Fig. 9. In the results, we can observe that a large number of predictions are assigned to the class normal pylorus, while a smaller number of predictions are assigned to the classes hemorrhoids and ulcerative colitis grade 1-2. However, these predictions are similar to that of the class-level accuracies of the ML model on the labeled data. Therefore, we can assume that the classes which achieved a high number of correct predictions on the labeled images are also more accurate on the unlabeled data. In contrast, it is hard to make any conclusions on the labels which had a low number of predictions as the models are not accurate enough. For future work, researchers could go trough the classifications of the unlabeled data and, for example, create a larger labeled dataset or perform failure analysis to find out why classes were confused or miss-classified. The class labels created during this experiments are available in the GitHub repository.

## Validation Summary

In the technical validation section, we provided baseline metrics and gave insight into the dataset’s statistical qualities to demonstrate it’s technical quality. With the large number of images available in *HyperKvasir*, we encourage other researchers to investigate and develop new and improved methods for the medical domain. This also includes an improved methodology for creating the ground truth in classes where there is a substantial inter-observer variation in the assessment, which might be used by other researchers to increase the number of labels and segmentations for the dataset.

## Usage Notes

In our research on detecting, classifying, and segmenting normal and abnormal findings in the GI tract, we have collected, to the best of our knowledge, the largest and most diverse dataset. These data are made available as a resource to the research community enabling researchers not only to have the ability to research the detection or classification of various GI findings but also differentiate between severity of the findings.

In short, we have used the labeled data to research the classification and segmentation of GI findings using both computer vision and ML approaches to potentially be used in live and post-analysis of patient examinations. Areas of potential utilization are analysis, classification, segmentation, and retrieval of images and videos with particular findings or particular properties from the computer science area. The labeled data can also be used for teaching and training in medical education. Having expert gastroenterologists providing the ground truths over various findings, *HyperKvasir* provides a unique and diverse learning set for future clinicians. Moreover, the unlabeled data is well suited for semi-supervised and unsupervised methods, and, if even more ground truth data is needed, the users of the data can use their own local medical experts to provide the needed labels. Finally, the videos can in addition be used to simulate live endoscopies feeding the video into the system like it is captured directly from the endoscopes enable developers to do image classification.

The dataset includes a series of scripts and text files that aim to help researchers quickly get started using the dataset for standard ML tasks such as classification. These are available at the GitHub repository for the dataset: http://www.github.com/simula/hyper-kvasir. Moreover, we provide three official splits of the dataset that can be used for cross-validation experiments. Keeping splits consistent between methods helps maintain a fair comparison of results. The scripts used to generate the plots, split data into different folds, and generate annotation files are included for reproducibility and transparency. These files may also be used to further experiment with the dataset. Finally, we include the files used to create our preliminary experiments.

There is currently a lot of research being performed in the field of GI image and video analysis, and we welcome and encourage future contributions in this area. This is not limited to using the dataset for comparisons and reproducibility of experiments, but also publishing and sharing new data in the future.

## Data Availability

In addition to releasing the data, we also make available the code used in the experiments. All code and additional data required for the experiments are available on GitHub at http://www.github.com/simula/hyper-kvasir.
